# MOFChecker: a package for validating and correcting metal–organic framework (MOF) structures

**DOI:** 10.1039/d5dd00109a

**Published:** 2025-05-08

**Authors:** Xin Jin, Kevin Maik Jablonka, Elias Moubarak, Yutao Li, Berend Smit

**Affiliations:** a Laboratory of Molecular Simulation (LSMO), Institut des Sciences et Ingénierie Chimiques, École Polytechnique Fédérale de Lausanne (EPFL) Switzerland berend.smit@epfl.ch

## Abstract

Metal–organic frameworks are promising porous materials for applications like gas adsorption, separation, transportation, and photocatalysis, but their large-scale computational screening requires high-quality, computation-ready structural data. Existing databases often contain errors due to experimental limitations, including inaccurately determined hydrogen positions, atomic overlaps, and missing components. We introduce MOFChecker to address these issues, providing tools for duplicate detection, geometric and charge error checking, and structure correction. Some errors can be systematically corrected through atomic adjustments on structures in the database, including deleting duplicated structures and adding missing hydrogen atoms, counterions, and linkers. Evaluation of established MOF databases, like the CoRE2014 database, indicates that 38% of structures contain significant errors, highlighting the importance of MOFChecker in ensuring accurate structural data for subsequent density functional theory (DFT) optimizations and computational studies. This work aims to enhance the reliability of MOF databases for high-throughput screening and practical applications.

## Abstract 2

MOFChecker, a package for MOF duplicate detection, geometric and charge error checking, and structure correction.

## Graphical abstract

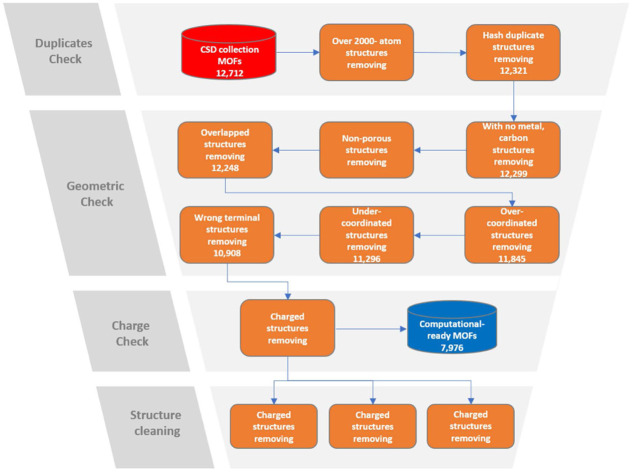

## Introduction

Metal–organic frameworks (MOFs) are crystalline materials with metal nodes and organic linkers. Their tunable chemical and structural characteristics make this an interesting class of materials for various applications, including gas adsorption and separation technologies.^1^ However, experimental testing across many MOF structures is impractical, creating a demand for high-throughput computational screening.^2^ This large-scale computational screening requires a database with crystallographic information for each MOF structure in a computation-ready format. Currently, several MOF databases have been reported for high-throughput computational screening, including the CoRE2014,^3^ CoRE2019,^4^ and CSD MOF collection^5^ databases, which contain experimentally derived structures. Databases with computational predicted structures include TobaCCo,^6^ Fernandez *et al.*,^7^ Majumdar *et al.*^8^ The OpenDAC2023 (ref. 9) and QMOF^10,11^ databases consist of a hybrid of both experimental and *in silico* structures.

Most databases used in computational studies source their data from the CSD. In most cases, a structure deposited in the CSD cannot be used directly in a computational study. For example, experimentally, it is difficult to determine the positions of hydrogen atoms accurately. It is common practice to report atomic occupancies that are not equal to one as overlapping atoms with fractional occupancy. In addition, the structures are often reported to have solvent molecules in the pores. The CoRE-MOF databases aimed to address these issues and provide the community with a computational-ready version of the MOF structures reported in the CSD.

One should not underestimate the complexity of “cleaning” these experimental structures. For example, one may introduce a charge when removing the solvent molecules. Often, this remains undetected; consequently, the corresponding metal node is assumed to be neutral. Many charged MOFs have been generated as these metal nodes are subsequently used to create *in silico* structures.

This is further compounded by the accidental deletion of essential structural components during solvent removal to get clean structures with open metal sites. It is worth noting that although most databases emphasize the importance of ensuring structural accuracy, many structures still have various problems.^12–14^ For example, White *et al.*^14^ have shown that many structure databases contain charged structures. A golden rule is to check the structures from the original publications one by one. Indeed, this is what one should do for a detailed study of a limited number of materials. However, for large-scale screening studies, this becomes quickly impractical.

This work introduces MOFChecker, an algorithm designed for MOF structure curation. It includes duplicated structure screening, geometric error checking, and charge error checking in MOF structures. Based on the check result, we also provide a workflow to clean and heal the structures with geometric or charge errors. This workflow typically repairs 50% of the problematic structures.

MOFChecker aims to ensure that all validated structures can be optimized into reasonable configurations based on a standard DFT optimization workflow, such as lsmo-AiiDA,^15^ thereby supporting subsequent high-throughput screening calculations.

The criterion for identifying erroneous structures in MOFChecker is whether they can achieve a reasonably optimized structure using DFT. This criterion is chosen to avoid potential structural distortions within reasonable limits, such as relatively too long or short bond length and distorted angle from the standard hybridization model. These structures can be fixed through DFT optimization, so it would be unfair to classify them as errors arbitrarily. However, determining whether a structure is a potential candidate for DFT optimization is challenging. We have established a set of error assessment tasks in MOF structures. This type of classification check has already been adopted by the newly published 2025 CoreMOF database.^16^ If one MOF structure passes the geometric structure check and charges check simultaneously, we assume it is ready for the subsequent DFT-level calculations.

## Methodology

### Geometric structure check

We have established a set of checking tasks based on common issues observed in MOF structures reported in different databases. These criteria are summarized in Table 1.

**Table 1:** Hash analysis and geometric structure check tasks. The significance of the evaluation criteria is categorized into three levels: (1) critical: failure indicates a definite structural error; (2) moderate: usually does not affect correctness but requires manual verification; (3) low: non-essential, primarily for functional checks

Task name	Description	Output	Significance
**Hash analysis**			
Formula	The chemical formula of MOF structure	String	Low
scaffold_hash	Identical hashes for connection graph	Hash string	Low
graph_hash	Identical hashes for graph including elements	Hash string	Low

**Geometric structure analysis**			
Density	The density of MOF structure	Float	Low
has_3d_connected_graph	If MOF structure is 3D connected	T/F	Moderate
is_porous	If MOF structure is porous	T/F	Critical
has_carbon	If MOF structure has C element	T/F	Critical
has_hydrogen	If MOF structure has H element	T/F	Moderate
has_metal	If MOF structure has metal element	T/F	Critical
has_atomic_overlaps	If MOF structure has atomic overlaps	T/F	Critical
has_lone_molecule	If MOF structure has isolated free molecule	T/F	Moderate
has_overcoordinated_c	If MOF structure has over-coordinated C atoms	T/F	Critical
has_overcoordinated_n	If MOF structure has over-coordinated N atoms	T/F	Critical
has_overcoordinated_h	If MOF structure has over-coordinated H atoms	T/F	Critical
has_undercoordinated_c	If MOF structure has under-coordinated C atoms	T/F	Critical
has_undercoordinated_n	If MOF structure has under-coordinated N atoms	T/F	Critical
has_suspicious_terminal_oxo	If MOF structure has O–M–O terminal	T/F	Critical
has_high_charges	If atoms have over +4 EQeq charge	T/F	Moderate

**Fig. 1:**
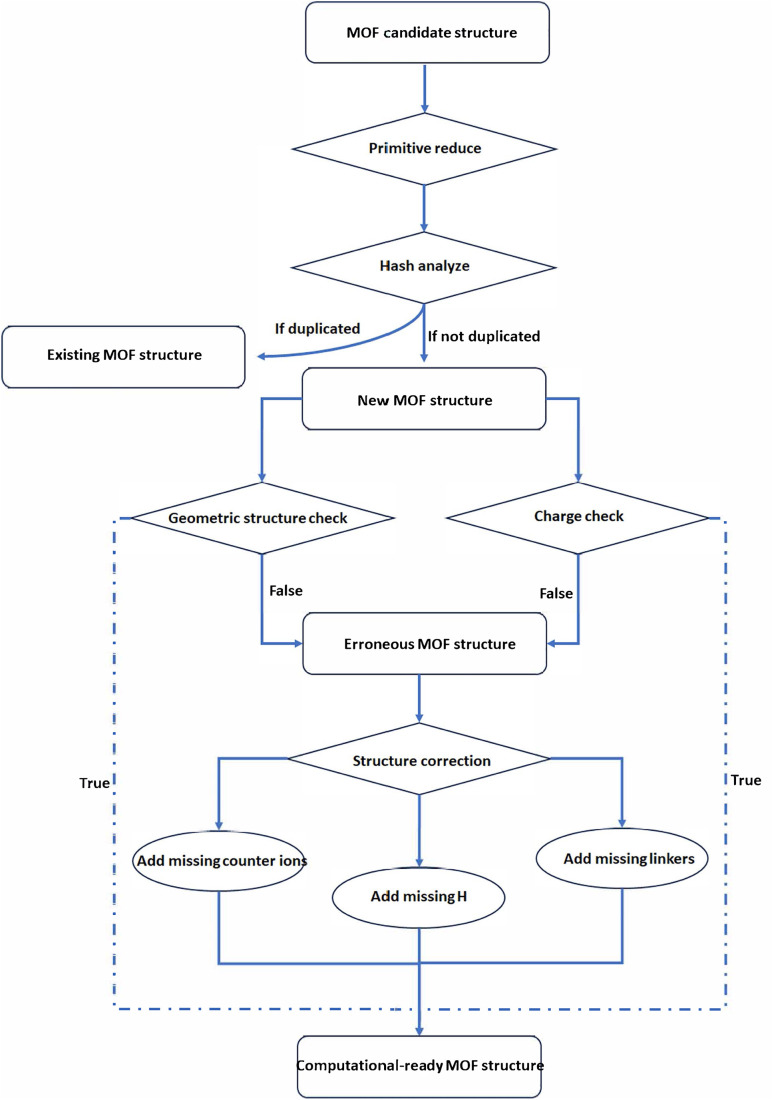
The workflow of MOFChecker, including validating and correcting MOF structures.

The schematics of the MOFChecker is shown in Fig. 1. The core architecture of the MOFChecker package is built on the Pymatgen library.^17–19^ It reads MOF structures from CIF files and generates the corresponding structure graph to perform structural checks.

First, we performed a hash analysis on the MOF structures, allowing for the detection of duplicates and structural similarities across the database.^20^ The scaffold hash (scaffold_hash) is unique for a given connectivity, independent of the atomic species in the structures. While the structure graph hash (graph_hash) also considers the atomic elements in the graph. The main workflow is: (1) reduce the structure to the primitive cell using Pymatgen and spglib; (2) analyze the bonding network based on VESTA cutoffs for bond distance^21^ and create a corresponding structure graph using Pymatgen; (3) compute the Weisfeiler–Lehman hash of the structure graph using networkx.^22^ In addition, by checking the dimensionality of the structure graph, we can also determine if it is a 3D MOF structure (has_3d_connected_graph).

Then, we apply Zeo++ for the porosity check (is_porous).^23^ Potential porosity is critical for many MOF applications, such as gas adsorption, separation, and transportation. We used a pore limiting diameter of more than 2.4 Å, approximately a hydrogen molecule's van der Waals diameter, as the threshold for distinguishing porous and non-porous MOFs, like the CoRE MOF database.

Metal nodes and organic linkers are also considered a critical defining criterion for MOFs. Before performing a detailed inspection of the structures, we filtered out those without carbon or metal elements (has_carbon and has_metal). The absence of hydrogen (has_hydrogen) is acceptable in cases where the ligands are all CN^−^.

**Fig. 2:**
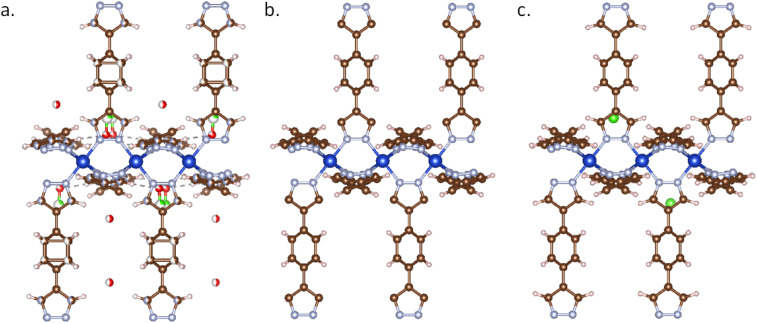
MOF structure of AFOYOK: (a) AFOYOK structure from CCDC database search, which has floating solvent molecules, atom overlaps (atom occupancy < 1); (b) AFOYOK structure from the CoRE2019 database, where all solvent molecules were removed (ASR structure), resulting in missing H atoms and counter ions; (c) AFOYOK structure curated by MOFChecker.

As demonstrated in Fig. 2, most CIF files obtained from experiments have a common issue: atoms are partially occupied. This “disorder” indicates the possibility of multiple spatial distributions of the structure. If a duplicate of the partially occupied atoms, such as aromatic rings, is not removed, it can result in atomic overlaps and over-coordination. There are also unbound floating solvent molecules, such as water molecules, often lacking explicit hydrogen atoms. To detect these issues, the atom overlaps (has_atomic_overlaps) is defined as the case where the distance between two adjacent atoms is less than the covalent radius of either atom. The isolated free molecule (has_lone_molecule), usually the free solvent or counter ions in the MOF structures, is defined as isolated molecules without connection with the frameworks by get_subgraphs_as_molecules function in pymatgen.

### Local chemical environment

The most challenging issue is verifying the correctness of the local environment structure. Due to the variability of the coordination environment at metal sites and the differing numbers of coordinated solvent molecules depending on structural preprocessing, we focus solely on verifying the organic ligands. The local environment of the metal sites is evaluated by ensuring charge balance, which is the task for the charge check. The over-coordinated carbon (has_overcoodinated_c) is defined as the case where the coordination number is more than 4. The over-coordinated hydrogen (has_overcoodinated_h) is defined as the case where the coordination number is more than 1. Nitrogen is treated differently due to its ability to coordinate with metals. Over-coordinated nitrogen (has_overcoodinated_n) is defined as having a coordination number greater than 4 with non-metal atoms.

We analyze under-coordinated atoms using classical valence bond theory. Admittedly, a single carbon valence bond analysis is too rigid, based solely on bond lengths and angles. It cannot accurately reflect the oxidation state properties, particularly for metals with variable oxidation states. However, it is highly effective for checking common organic ligands, as they have relatively well-defined and standard bond lengths and bond angle ranges. It is important to note that our focus here is solely on the structural accuracy of the organic ligands without considering the metal charge properties. Specifically, we first check the non-metal coordination number of the atom to predict its hybridization. Then, based on this predicted hybridization, we examine whether the structure conforms to the expected topological configuration by checking bond lengths and angles. If it does not, we analyze whether the deviation is due to structural errors like under-coordination, over-coordination, or the extra charge at this site.

We first analyze the number of non-metal connections to check under-coordinated carbon atoms (has_undercoodinated_c). If the non-metal connection number equals 1, it is identified as a C–N triple bond or a C–C triple bond by checking bond networks. If the non-metal connection number equals 2, without considering the case of carbon anions, it is identified as a sp^1^ hybridization, which should be a linear configuration. Finally, for a non-metal connection number equal to 3, without considering the case of carbon anions, it is identified as a sp^2^ hybridization, which should be a configuration where the atom is coplanar with its adjacent atoms.

The check for nitrogen (has_undercoodinated_n) is much more complex. Carbon anions rarely occur in MOF environments, whereas nitrogen sites may carry a negative charge. If the non-metal connection number equals 1, it is identified as either a C–N triple bond or an N–N triple bond. If the non-metal connection number equals 2, without considering N anions, it is either a sp^1^ hybridization or sp^2^ hybridization; we also check whether the N atom is coplanar with its adjacent atoms. If there is a negative charge at the N site, which is reasonable from the point of view of geometric structures, we do not label such nitrogen atoms as under-coordinated; instead, this issue is deferred to the subsequent charge check.

For oxygen atoms, due to their versatile bonding configurations, it is generally possible to identify a reasonable valence bond arrangement from a geometric structure perspective. Therefore, we similarly defer their evaluation to the charge check. It is worth noting a specific suspicious case where an oxygen atom acts as a terminal atom and is bonded to only one metal (has_suspicious_terminal_oxo). This error is standard in cases where water molecules are coordinated, but the hydrogen atoms are not correctly displayed. However, in uranium metal nodes, as well as in osmium ones, such oxygen terminal structures can exist. This flag can be incorrectly flagged, and users have to check the structures manually. Over-coordination and under-coordination of other elements are determined based on their connections to C, N, and O atoms.

Identifying under-coordinated atomic positions and possible N anions is important for checking the geometric structure and adding hydrogen atoms in the structure correction process.

### Charge check

As mentioned before, the functional form of the bond valence method might sometimes be too rigid, as it is based solely on bond lengths. Technically, this can cause problems when considering experimental and force field relaxed structures or when the bond lengths have not been determined accurately. If we focus on the bonding environment at the metal site, like MOSAEC,^14^ we can certainly obtain the impossible oxidation state of metal when the structure has charge errors. However, merely identifying charged structures is not enough; we also need to determine the exact charge of each structure for use in the subsequent correction process.

The workflow for determining the charge in MOFChecker is divided into three main steps: (1) determine the oxidation states of the metal sites; (2) identify the positive charges introduced by non-metal components, such as counter cations; (3) identify the negative charges introduced by non-metal components, such as organic ligands. A neutral MOF structure should have zero net charge.

First, we apply OxiMACHINE^24^ to determine the oxidation states of the metal sites. OxiMACHINE is a pre-trained machine-learning model that assigns integer oxidation states based on the metal's local chemical environment. Since the model is trained on structures from the CSD, which includes many structures with missing hydrogen atoms, missing charged solvents, counter-ions, and atomic overlap issues, it can effectively assign oxidation states to metal atoms regardless of whether the structure is correct. It does not require manual definition of the metal node components, and it can directly predict the oxidation state of metal atoms within the local environment of the MOF.

The positive charges introduced by non-metal components usually come from oxonium cations and ammonium cations. Based on the structure graph in the previous section, we can easily search those structures by calculating the non-metal coordination number. For the oxonium ion, the coordination number of oxygen is 3; for the ammonium ion, the coordination number of nitrogen is 4.

We separate the organic ligands and metals when accounting for charges. This allows us to disregard the influence of metal coordination when analyzing the local chemical environment of atoms. However, determining the negative charges introduced by organic ligands is still challenging because of their diversity and complexity. We propose using an inductive approach, in which we systematically identify atomic sites likely to carry formal charges. These sites are most commonly associated with halogen, nitrogen, oxygen, and sulfur atoms. In this context, formal charge denotes an integer value conventionally assigned to an atom. This charge is conceptually distinct from the actual electronic charge distribution derived from methods such as EqEq or DDEC. The formal charge is a simplified descriptor and does not necessarily reflect the true charge delocalization within the linker framework. This approach offers the advantage of simplifying the overall analysis by categorizing the problem into a set of manageable cases. For example, in phosphorus-containing acidic ligands, various species such as phosphate, phosphite, hypophosphite, and phosphonic acid derivatives may arise depending on the oxidation state of phosphorus. In addition, these linkers contain different numbers of protons, which in turn also influence the overall charge. However, by focusing solely on formal charges, one finds that the charge is localized exclusively on oxygen atoms, independent of the specific phosphorus oxidation state. Actually, the formal charge on oxygen atoms is particularly straightforward to enumerate, with the help of the bond networks established in the previous structural check, because oxygen typically forms bonds with no more than two non-metal atoms. Some other ligands, such as sulfonate linkers and perchlorate groups, where the formal charge is likewise localized on oxygen atoms, regardless of the central atom's oxidation state. In our classification, these cases are divided into oxygen groups.

Once we have obtained the values for the three types of charges, we calculate the MOF structure's net charge. If the net charge is 0, the structure passes the charge check. If it is not 0, the structure proceeds to the subsequent correction process based on the calculated net charge.

## Structure correction

It is of great importance to perform structure correction in MOF database curation. We identify specific errors in those problematic structures through detailed and comprehensive checks. For duplicates, non-MOF structures, and non-porous structures can be directly removed. For MOFs with geometric structure errors, such as atomic overlaps or over-coordinated atoms, we also output the coordinates of the problematic atom positions. Users may manually remove these atoms depending on their specific requirements. For under-coordinated atoms, the correction process involves identifying these atoms as priority sites for modification. Typically, hydrogen atoms are added to these sites to restore chemical completeness and ensure structural validity.

For charged structures, we categorize the issues into three main types: missing counter-ions, missing hydrogen atoms, and missing charged linkers. The principle of correction is to restore the structure as closely as possible to the one reported in the original literature.

By applying the CSD Python library API, we retrieve the chemical formula of the original MOF structure. It allows us to determine whether the source of errors originated from missing counter-ions by comparing it with the chemical formula of the current CIF file. If missing counter-ions are ruled out, we determine the nature of the problem by analyzing the net charge of the structure. A positive net charge suggests missing hydrogen atoms, while a negative net charge indicates missing charged linkers. For *in silico* MOFs, if it is checked as a charge error, we need to return to the building blocks to restore it.

### Missing counter ions

It is common to include unbound solvent molecules and counterions in experimentally obtained MOF structures. These neutral solvents often need to be removed for a computation-ready database. However, the charge distribution will be significantly affected if one (mistakenly) deletes counter ions or charged solvent molecules without additional attention in subsequent calculations. Researchers typically assume such structures to be neutral, leading to an adjusted charge distribution to compensate for the unbalanced charges, which can drastically affect the accuracy of the calculation results.

**Fig. 3:**
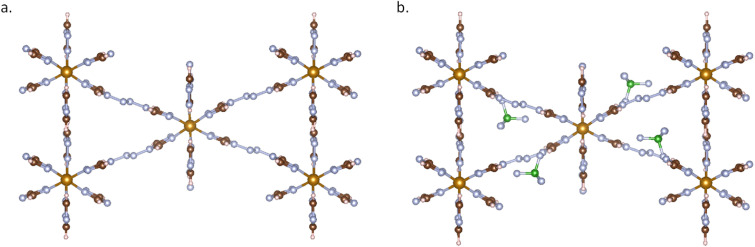
MOF structure of BAXFUD: (a) BAXFUD structure (ASR structure) from the CoRE2019 database, where have missing counter ions BF_4_^−^; (b) The BAXFUD structure was curated by MOFChecker with added counter ions.

Using MOFChecker, we can identify the amount of missing charge in the structure. Users can determine the counterions used in the original literature by calling the CSD library API or get the complete chemical formula directly from the CCDC database entry. Then we can perform Monte Carlo (MC) simulations under the NVT ensemble using the Universal Force Field (UFF) to reinsert these missing counterions. Fig. 3 gives an example of a MOF that was originally charged. By adding the missing counter ions (BF_4_^−^) a neutral MOF is obtained.

It is important to note that during MC simulations, the charge distribution of the MOF structure is typically required. However, as discussed earlier, accurately describing the charge distribution of these charged systems is challenging and time-consuming. Considering that absolute accuracy in ion distribution is not critical at this stage—since the structure will undergo further optimization in subsequent DFT calculations—we can perform fast MC simulations without accounting for Coulomb interactions to insert the missing ions efficiently.

It is important to note that the MOF structure's porosity may change after adding ions. This requires reevaluation to ensure that the structure meets the requirements for subsequent property calculations.

### Missing H atoms

In experiments, XRD often fails to determine the positions of hydrogen atoms accurately, and most hydrogen atoms are added during post-processing. For nitrogen and oxygen, especially those coordinated to metals, hydrogen atoms are often not correctly added. This leads to incorrect assessments of the charges on N and O atoms. As mentioned in the last section, if not correctly annotated, such charge inaccuracies can result in a mistaken assumption of the MOF structure's charge neutrality, subsequently impacting the accuracy of the charge distribution in computational studies.

**Fig. 4:**
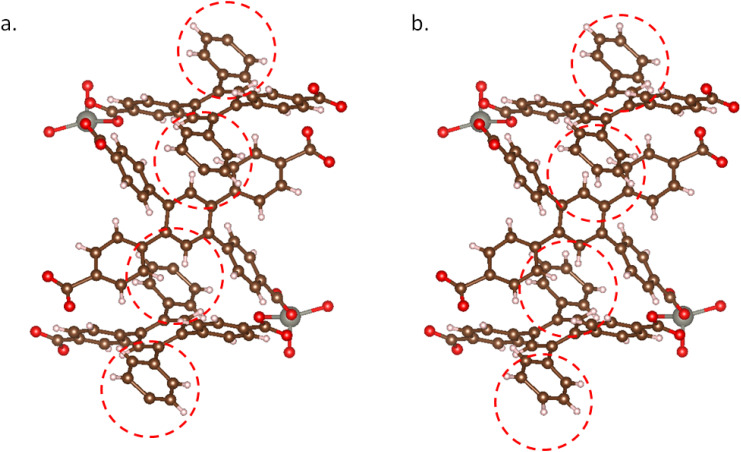
MOF structure of MIFKUJ: (a) MIFKUJ structure (ASR structure) from the CoRE2019 database, where have missing H atoms; (b) MIFKUJ structure curated by MOFChecker with adding H atoms.

Using MOFChecker, we can determine the number of missing charges corresponding to the number of hydrogen atoms that need to be added. The sites for hydrogen addition are prioritized based on the highest conjugate acid p*K*_a_ values. Specifically, during the charge check, hydrogen atoms are sequentially added to the following sites: under-coordinated C sites, O^2−^ ion sites, nitrogen anion sites, O^−^ ion sites, and neutral O atom sites (see Fig. 4 for an example).

### Missing charged linkers

**Fig. 5:**
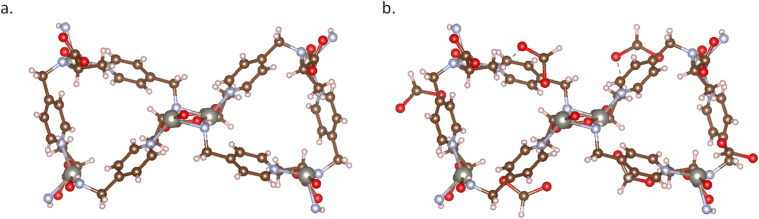
MOF structure of BEPLUF: (a) BEPLUF structure (ASR structure) from the CoRE2019 database, where have missing formate linkers; (b) BEPLUF structure curated by MOFChecker with adding formate linkers.

In computation-ready databases, activation of MOF materials typically involves removing neutral solvent molecules coordinated to metal sites, such as water or DMF. However, in some cases, the removed solvent molecules are charged, or coordinated counter-ions, such as formate or nitrate, are inadvertently deleted (for an example, see Fig. 5).

Using MOFChecker, we can identify the missing charge information and, by comparing it with the CSD library, determine the types of removed solvent molecules. We also provide the code that supports the addition of single-coordinated solvent molecules at the open metal site. Multi-coordinated solvent molecules are not considered, as their removal would indicate that the MOF structure has already been significantly altered.

## Results and discussion

**Fig. 6:**
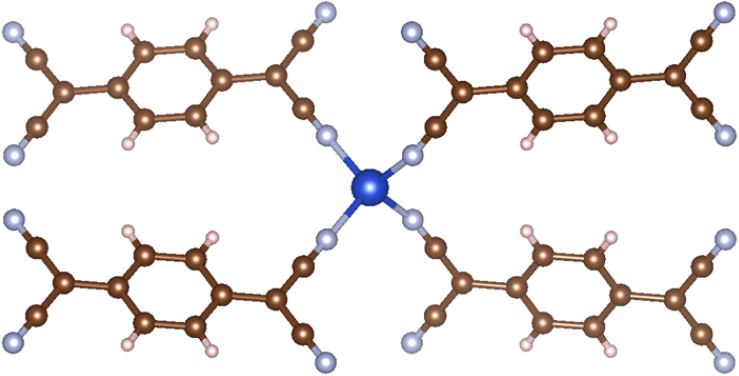
MOF structure of CUSZIB, which has carbon anions structure.

At this point, it is important to note that we do not expect the MOFChecker to be perfect for all MOFs. To obtain some statistics on the percentage of false positives and negatives, we manually examined 200 randomly selected structures predicted to be correct. We achieved an accuracy rate of 97.5%, implying that we have 2.5% false positives. We also manually checked 300 randomly selected structures predicted to be incorrect, divided into three sets with 100 MOFs each: a set with only geometric structure errors, one with only charge errors, and one with both errors. The accuracy rate for this group was 92%. Hence, having 8% false negatives. The analysis of incorrect predictions reveals the following potential causes: (1) deviation in OxiMACHINE predictions. The oxidation state predictions from OxiMACHINE deviate from the correct values, particularly for metals with variable oxidation states, such as Co(ii/iii) and Mn(ii/iii); (2) neglected carbon anions. Carbon anions were not considered in the checking. Typically, such structures are stabilized by conjugated systems with strong electron-withdrawing groups at the α-position, such as the CUSZIB MOF structure, as shown in Fig. 6.

**Fig. 7:**
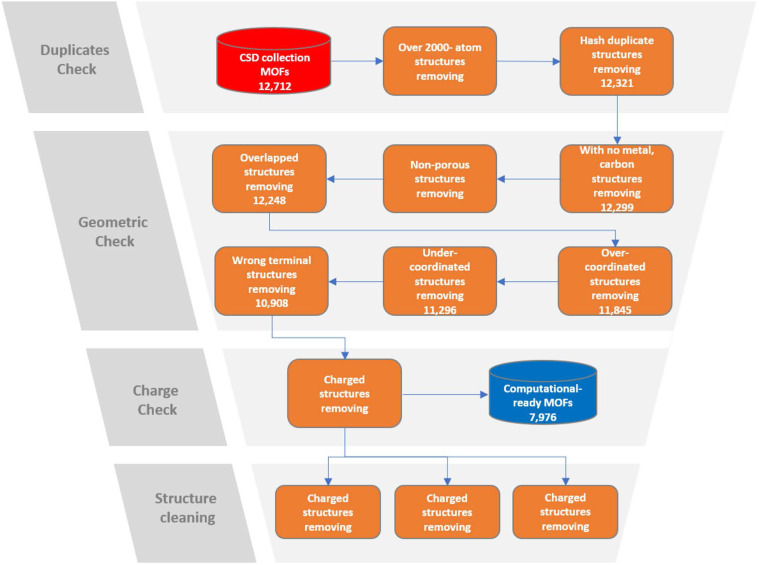
Structure checking workflow on CSD collection database.

Upon evaluating the well-known CoRE2014 MOF database with MOFChecker, we found that 11.8% of the structures failed the geometric structure check, and 32.5% failed the charges check. Approximately 38.0% of the structures did not pass at least one of the criteria. We also evaluated the CSD MOF collection database as shown in Fig. 7. In this case, 11.5% of the structures failed the geometric structure check, and 31.5% failed the charges check. In total, approximately 35.3% of the structures did not pass the checks. This result is not surprising, as many of those structures are from the same source.

In contrast, a sample from the QMOF database showed that most of the structures passed the MOFChecker test. This is not surprising as the QMOF database has been carefully curated and optimized with DFT; structures that failed to pass the MOFChecker test will also fail in DFT simulation.

It is possible to do DFT calculations with a charged structure. However, if one calculates binding energy in such a charged structure, one would expect a significantly overestimated binding energy. Indeed, Sriram *et al.*,^9^ computed binding energies of CO_2_ larger than 100 kJ mol^−1^, while the binding energy of the corresponding neutral structure would be less than 60 kJ mol^−1^.

**Fig. 8:**
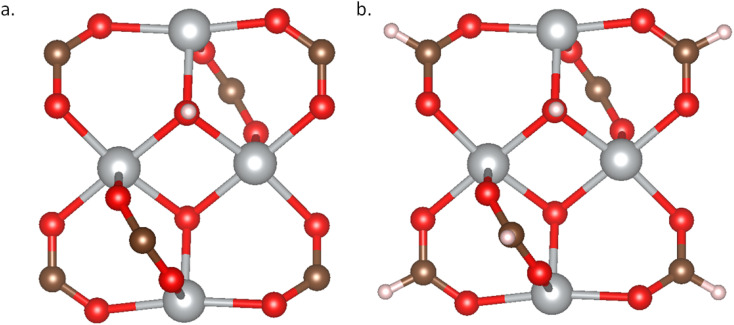
Metal node fragment. (a) Structure of mn1_LITCIC; (b) structure of mn1_LITCIC with H atom terminated connection point.

In addition to experimental databases, *in silico* MOF databases face the same issues. The fragments of these *in silico* MOFs, including metal nodes and organic linkers, usually originate from either the CoRE database or the CSD database. Like experimental databases, these structures must undergo rigorous structure checks, charge validation, and DFT optimization processes to ensure their reliability for further use in research and applications. We adopt two methods to analyze the structures from *in silico* databases by checking the geometric structure and charge of the fragments and the MOF structures. For each fragment, we need to terminate one H atom at the connection site to avoid introducing extra under-coordinated atoms, as shown in Fig. 8.

In the database of Majumdar *et al.*,^8^ we individually conducted geometric structure and charge checks on the metal nodes and MOF structures. Among the 14 metal nodes, two nodes were found to be erroneous. However, when checking nearly 1300 MOF structures, it was revealed that 38% of the structures contained errors. The lack of force field optimization may introduce extra structure distortion. Unlike MOF structures in experiments with optimal cell parameters, even with a more accurate force field, like MACE optimization,^25^ there are still unavoidable geometric structure errors without performing any cell optimization calculation for *in silico* MOFs. However, this kind of error is acceptable because it can be fixed by following DFT cell optimization and geometry optimization, as long as we ensure the correctness of building blocks.

## Concluding remarks

MOFChecker provides a reliable workflow for validating and correcting MOF structures. By addressing common issues such as geometric inaccuracies, charge imbalances, and incomplete structural data, MOFChecker helps to ensure that MOF databases meet the stringent quality requirements necessary for high-throughput computational studies. It enables comprehensive checks and systematic corrections of problematic structures. Our evaluation of widely used MOF databases demonstrates the prevalence of errors, with around 38% of the structures failing at least one validation criterion. These findings underscore the critical need for robust curation tools to avoid propagating errors. The implementation of MOFChecker enhances the reliability of MOF databases. It facilitates their integration into DFT workflows, thus accelerating the discovery and optimization of MOFs for practical applications.

## Conflicts of interest

There are no conflicts to declare.

## Data Availability

The checking results of several databases can be found in detail on Zenodo https://zenodo.org/records/14844662. The MOF structures can be checked using the MOFChecker Python package https://github.com/Au-4/mofchecker_2.0. The MOFChecker code used in this work has been archived and frozen on Zenodo https://zenodo.org/records/15341714.
